# Quality of Life and Its Determinants Among Individuals With Diabetes in Saudi Arabia: A Cross-Sectional Online Survey

**DOI:** 10.7759/cureus.69394

**Published:** 2024-09-14

**Authors:** Najlaa M Alsudairy, Sarah M Alzaidi, Afnan G Alghamdi, Mona S Alrashidi, Deemah A Altashkandi

**Affiliations:** 1 Department of Family Medicine, National Guard Hospital, Jeddah, SAU

**Keywords:** diabetes mellitus, emotional health, quality of life, saudi arabia, social health

## Abstract

Background

Diabetes mellitus is a significant public health issue globally, with increasing prevalence rates. In Saudi Arabia, a substantial proportion of the adult population is affected. While the impact of diabetes on physical health is well-documented, less is known about its effects on emotional and social well-being in this region.

Methods

From January to March 2024, we conducted a cross-sectional online survey of 207 individuals with diabetes in Saudi Arabia. The survey, available in Arabic and English, assessed physical symptoms, emotional health, social interactions, and overall quality of life. Descriptive statistics were used for analysis.

Results

The majority of participants were aged 31-45 years (n=63, 30.4%) and female (n=118, 56.9%). Type 2 diabetes was the most common (n=122, 59.1%). Regarding physical health, 34.6% (n=72) rated their health as good while 37.2% (n=77) experienced physical symptoms sometimes and 17.9% (n=37) often. Emotional health challenges were reported by 39.2% (n=81) experiencing anxiety and 33.9% (n=70) experiencing depression. Social interactions were impacted, with 28.6% (n=59) reporting challenges in relationships and 14.3% (n=30) facing significant impacts on recreational activities. Overall quality of life was rated as good by 40.4% (n=83), fair by 29.9% (n=62), and very poor by 4.8% (n=10).

Conclusions

The study highlights the significant effects of diabetes on the physical, emotional, and social aspects of life in Saudi Arabia. The findings underscore the need for comprehensive care strategies that incorporate medical treatment, emotional support, and social integration to improve the overall quality of life for individuals with diabetes.

## Introduction

Diabetes mellitus represents a significant public health challenge globally, with its prevalence steadily increasing across diverse populations. In Saudi Arabia, the prevalence of diabetes has reached alarming levels, with recent estimates indicating that approximately 25% of the adult population is affected by this chronic condition [1-4]. Diabetes imposes a considerable burden on individuals, healthcare systems, and societies at large, influencing not only physical health but also emotional and social well-being [2,4].

The impact of diabetes extends beyond the management of blood glucose levels and the prevention of acute complications. Individuals with diabetes often experience a range of chronic physical symptoms, including fatigue, pain, and impaired mobility, which can significantly affect their daily lives and overall physical health. Furthermore, the psychological burden associated with managing a chronic condition like diabetes is substantial. Many individuals with diabetes report high levels of anxiety and depression, which can further complicate disease management and contribute to a diminished quality of life [2-9].

Social health is also critically affected by diabetes. Individuals may encounter challenges in maintaining social relationships and participating in recreational activities due to their condition. These social dimensions of diabetes can contribute to feelings of isolation and affect overall well-being, highlighting the need for comprehensive support systems that address not only medical needs but also emotional and social aspects [10,11].

Despite the known impacts of diabetes, there is a relative paucity of research focusing specifically on the quality of life among individuals with diabetes in Saudi Arabia. Most existing studies have concentrated on clinical outcomes or specific complications, with limited attention given to the broader aspects of patient well-being. Understanding how diabetes affects individuals' daily lives, emotional states, and social interactions is crucial for developing effective interventions and support strategies [5-12].

This study aims to fill this gap by providing a detailed assessment of the quality of life among individuals with diabetes in Saudi Arabia. By evaluating various dimensions of health-including physical symptoms, emotional well-being, social challenges, and overall quality of life research seeks to offer valuable insights that can inform better clinical practices and support mechanisms. Addressing these issues comprehensively will contribute to improving the overall care and support for individuals living with diabetes, ultimately enhancing their quality of life and health outcomes.

## Materials and methods

Study design and setting

This cross-sectional study was conducted via an online survey to assess various aspects of quality of life among individuals with diabetes in Saudi Arabia. The survey was distributed from January to March 2024 through social media platforms, diabetes support groups, and healthcare provider networks. Participants were invited to complete the survey voluntarily, and informed consent was obtained from all respondents before they began the survey. The choice of an online platform was intended to maximize reach and convenience for a diverse participant pool.

Participants

The study sample consisted of 207 individuals diagnosed with diabetes. To qualify for inclusion, participants had to be at least 18 years old and have a confirmed diagnosis of diabetes. Individuals who did not meet these criteria or who were unable to provide informed consent were excluded from the study. Demographic information collected included age, gender, type of diabetes, and duration of diagnosis, allowing for a comprehensive analysis of the sample population.

Survey instrument

The survey instrument was carefully developed based on a review of existing literature and consultation with experts to ensure it addressed relevant aspects of diabetes management and quality of life. The survey comprised several sections, including demographics, physical health, emotional health, social health, and general well-being. Questions were designed to capture data on participants’ age, gender, type of diabetes, and the duration of their diagnosis. Additionally, the survey assessed the frequency and impact of physical symptoms related to diabetes, overall physical health, levels of anxiety and depression, satisfaction with healthcare support, challenges in maintaining social relationships, and comfort in discussing diabetes. The overall quality of life and perceived impact of diabetes on well-being were also evaluated, along with participants’ preferences for additional support.

Data collection

Data were collected using an online survey platform, which allowed for efficient and widespread distribution. The survey was available in both Arabic and English to accommodate the linguistic diversity of respondents. Participants accessed the survey through a unique link and completed it anonymously. The estimated time to complete the survey was approximately 10-15 minutes, ensuring that it was manageable for respondents and likely to yield high completion rates.

Data analysis

Data analysis was performed using descriptive statistics to summarize the responses. Frequencies and percentages were calculated for all categorical variables to provide an overview of the participants’ demographics and their responses to survey questions. The data were aggregated and presented in tabular form to facilitate the interpretation of key findings. Statistical software was employed to ensure precise and reliable analysis.

## Results

Demographics of survey respondents

A total of 207 individuals with diabetes participated in the survey. The majority of respondents were aged 31-45 years (n=63, 30.4%), followed by those aged 18-30 years (n=58, 28.1%). Participants over 60 years constituted 14.8% (n=29) of the sample. Gender distribution was fairly even, with 56.9% (n=118) identifying as female and 43.1% (n=89) as male. Type 2 diabetes was the most commonly reported type (n=122, 59.1%), followed by Type 1 diabetes (n=38, 18.5%). Approximately 16.7% (n=35) of respondents were unsure of their diabetes type. In terms of duration since diagnosis, 42.1% (n=87) had been diagnosed for 1-5 years while 15.9% (n=33) had lived with diabetes for over 10 years (Table 1).

**Table 1:** Demographics of survey respondents (N=207) The table presents the demographic characteristics of the survey respondents. Values are represented as n (%).

Demographic Variable		Number of Respondents	Percentage (%)
Age Group	Under 18	14	6.8%
	18-30	58	28.1%
	31-45	63	30.4%
	46-60	42	20.0%
	Over 60	29	14.8%
Gender	Male	89	43.1%
	Female	118	56.9%
Type of Diabetes	Type 1	38	18.5%
	Type 2	122	59.1%
	Gestational	12	5.8%
	I don’t know	35	16.7%
Duration of Diabetes Diagnosis	Less than 1 year	22	10.6%
	1-5 years	87	42.1%
	6-10 years	54	26.0%
	More than 10 years	33	15.9%

Physical health of participants

Regarding the frequency of physical symptoms related to diabetes, 37.2% (n=77) of respondents reported experiencing symptoms sometimes, and 17.9% (n=37) reported experiencing them often. A total of 34.6% (n=72) rated their overall physical health as good in the past month, whereas 32.7% (n=68) rated it as fair. Diabetes affected daily activities sometimes for 25.5% (n=53) of respondents and often for 12.0% (n=25) (Table 2).

**Table 2:** Frequency and impact of physical symptoms and overall physical health (N=207) The table details the frequency and impact of physical symptoms, as well as overall physical health ratings. Values are represented as n (%).

Physical Health Variable		Number of Respondents	Percentage (%)
Frequency of Physical Symptoms	Never	21	10.1%
	Rarely	47	22.6%
	Sometimes	77	37.2%
	Often	37	17.9%
	Always	19	9.2%
Overall Physical Health Rating	Excellent	29	13.9%
	Good	72	34.6%
	Fair	68	32.7%
	Poor	22	10.6%
	Very Poor	15	7.2%
Impact on Daily Activities	Not at all	45	21.7%
	Rarely	62	29.8%
	Sometimes	53	25.5%
	Often	25	12.0%
	Always	14	6.7%

Emotional health of participants

In terms of anxiety about diabetes management, 39.2% (n=82) of respondents felt anxious sometimes, and 16.3% (n=34) felt anxious often. Feelings of depression or sadness related to diabetes were reported sometimes by 33.9% (n=71) of respondents and often by 13.4% (n=28). Satisfaction with healthcare support varied, with 36.9% (n=77) being satisfied and 18.8% (n=39) very satisfied. However, 11.6% (n=24) were dissatisfied, and 6.8% (n=14) were very dissatisfied (Table 3).

**Table 3:** Emotional health regarding diabetes management (n=207) This table shows emotional health metrics related to diabetes management, including anxiety, depression, and satisfaction with healthcare support. Values are represented as n (%).

Emotional Health Variable		Number of Respondents	Percentage (%)
Anxiety about Diabetes Management	Never	27	12.9%
	Rarely	41	19.6%
	Sometimes	82	39.2%
	Often	34	16.3%
	Always	23	11.0%
Feelings of Depression/Sadness	Never	31	14.9%
	Rarely	52	24.8%
	Sometimes	71	33.9%
	Often	28	13.4%
	Always	12	5.8%
Satisfaction with Healthcare Support	Very Satisfied	39	18.8%
	Satisfied	77	36.9%
	Neutral	53	25.5%
	Dissatisfied	24	11.6%
	Very Dissatisfied	14	6.8%

Social health of participants

Challenges in maintaining social relationships due to diabetes were reported sometimes by 28.6% (n=60) of respondents and often by 11.9% (n=25). Regarding participation in social or recreational activities, 30.8% (n=65) reported a moderate impact, and 14.3% (n=30) reported a significant impact. Comfort in discussing diabetes with others was high, with 25.2% (n=53) being very comfortable and 34.3% (n=72) comfortable. However, 18.1% (n=37) reported feeling uncomfortable or very uncomfortable discussing their condition (Table 4).

**Table 4:** Social health and comfort in discussing diabetes (n=207) This table summarizes challenges in social relationships, participation in activities, and comfort in discussing diabetes. Values are represented as n (%).

Social Health Variable		Number of Respondents	Percentage (%)
Challenges in Maintaining Social Relationships	Never	35	16.8%
	Rarely	48	22.9%
	Sometimes	60	28.6%
	Often	25	11.9%
	Always	17	8.1%
Participation in Social/Recreational Activities	Not at all	32	15.3%
	A little	58	27.8%
	Moderately	65	30.8%
	Quite a bit	30	14.3%
	Extremely	15	7.1%
Comfort Discussing Diabetes	Very Comfortable	53	25.2%
	Comfortable	72	34.3%
	Neutral	41	19.5%
	Uncomfortable	22	10.5%
	Very Uncomfortable	16	7.6%

The general well-being of participants

The overall quality of life related to living with diabetes was rated as good by 40.4% (n=85) of respondents while 29.9% (n=63) rated it as fair. A small proportion rated their quality of life as very poor (4.8%, n=10). The perceived impact of diabetes on overall well-being was neutral for 38.6% (n=81) of respondents and negative for 17.1% (n=36). To improve quality of life, 32.4% (n=68) of respondents indicated a need for better medical treatment, 25.7% (n=54) sought more educational resources, and 27.6% (n=58) desired improved emotional support. Enhanced social support and other types of support were mentioned by 9.5% (n=20) and 2.9% (n=6) of respondents, respectively (Table 5).

**Table 5:** Overall quality of life and perceived impact of diabetes (N=207) This table outlines the overall quality of life, perceived impact of diabetes, and types of additional support needed. Values are represented as n (%).

General Well-Being Variable		Number of Respondents	Percentage (%)
Overall Quality of Life	Excellent	31	14.8%
	Good	85	40.4%
	Fair	63	29.9%
	Poor	20	9.5%
	Very Poor	10	4.8%
Perceived Impact on Well-Being	Very Positive	18	8.6%
	Positive	55	26.2%
	Neutral	81	38.6%
	Negative	36	17.1%
	Very Negative	14	6.7%
Additional Support Needed	Better medical treatment	68	32.4%
	More educational resources	54	25.7%
	Improved emotional support	58	27.6%
	Enhanced social support	20	9.5%
	Other (Please specify)	6	2.9%

## Discussion

This study provides a comprehensive evaluation of the quality of life among individuals with diabetes in Saudi Arabia, highlighting critical areas of concern and offering insights into the broader implications for diabetes management and support. The findings reveal a complex interplay between the physical, emotional, and social dimensions of health, which are pivotal for understanding the full impact of diabetes on individuals' lives [10-15].

Our results indicate that a significant proportion of respondents experience frequent physical symptoms related to diabetes, with 37.2% reporting symptoms sometimes and 17.9% often. This aligns with existing literature that emphasizes the burden of diabetes-related symptoms on daily functioning and overall health. The relatively high proportion of individuals rating their physical health as fair or poor underscores the need for effective management strategies and interventions. Addressing physical symptoms and improving overall health should be a priority in diabetes care to enhance patients' quality of life and mitigate the daily challenges associated with the condition [2-6].

The study highlights substantial emotional challenges faced by individuals with diabetes. A noteworthy percentage of respondents reported experiencing anxiety (39.2%) and depression (33.9%) related to their condition. These findings are consistent with previous research showing that diabetes management often entails significant psychological stress, which can adversely affect patients' mental health. The variability in satisfaction with healthcare support - 36.9% satisfied and 11.6% dissatisfied - suggests that there is room for improvement in the support provided by healthcare professionals. Enhancing emotional support and integrating mental health services into routine diabetes care could help address these issues and improve overall patient well-being.

The survey results reveal that a considerable number of participants face challenges in maintaining social relationships and participating in recreational activities due to diabetes. Specifically, 28.6% reported moderate challenges in social interactions, and 14.3% experienced significant impacts on their participation in activities. This underscores the social dimensions of diabetes management and the importance of addressing these aspects in patient care. Social support networks play a crucial role in managing chronic conditions, and improving social integration and support could alleviate some of the burdens faced by individuals with diabetes [4-9].

The majority of respondents rated their overall quality of life as good, yet 29.9% rated it as fair, and 4.8% rated it as very poor. This spectrum of responses reflects the diverse experiences of individuals living with diabetes and highlights the need for personalized care approaches. The perceived negative impact of diabetes on overall well-being, reported by 17.1% of participants, further emphasizes the need for comprehensive support strategies. Our findings suggest that interventions aimed at improving medical treatment, increasing educational resources, and enhancing emotional support could significantly benefit patients' quality of life [5-8].

Strengths and limitations

This study's strengths include its focus on a diverse sample of individuals with diabetes across Saudi Arabia and its use of a comprehensive survey instrument to assess various aspects of quality of life. However, several limitations must be acknowledged. The reliance on self-reported data may introduce biases, and the online nature of the survey might have excluded individuals with limited access to technology. Additionally, while the sample provides valuable insights, it may not fully represent all individuals with diabetes in Saudi Arabia, potentially limiting the generalizability of the findings.

## Conclusions

This study underscores the profound impact of diabetes on individuals' physical, emotional, and social well-being in Saudi Arabia. The findings reveal significant challenges in managing diabetes-related symptoms, emotional stress, and social interactions, highlighting the need for comprehensive and personalized care strategies. Addressing these multifaceted issues through enhanced medical treatment, better educational resources, and increased emotional and social support could substantially improve the quality of life for individuals living with diabetes. By adopting a holistic approach to diabetes management, healthcare providers can better support patients and foster a more effective and empathetic care environment.
